# Iris Pigmented Lesions: Unraveling the Genetic Basis of Iris Freckles and Nevi

**DOI:** 10.1167/iovs.66.4.62

**Published:** 2025-04-22

**Authors:** Julia Boldu-Roig, Elena Sorli-Clemente, Aida Kuljuh-Causevic, Alba Loras, Alfonso Anton, Conrado Martinez-Cadenas

**Affiliations:** 1 Department of Ophthalmology, Catalan Retina Institute, Barcelona, Spain; 2 Department of Ophthalmology, Castellón General University Hospital, Castellón, Spain; 3 Department of Medicine, Jaume I University of Castellón, Castellón, Spain

**Keywords:** iris pigmentary patterns, iris freckles, iris nevi, eye color, genetics

## Abstract

**Purpose:**

To investigate the diversity of pigmented benign lesions in the human iris, aiming to provide insights for forensic, biomedical, and ophthalmological research.

**Methods:**

A cohort of 1014 individuals of Spanish descent was analyzed. Digital slit-lamp photographs were used to evaluate iris pigmentation traits, including iris freckles, iris nevi, iris color, and the presence of a pigmented collarette. A candidate gene association study was performed on these pigmentation traits.

**Results:**

Both iris freckles and nevi were associated with increased age, female sex, pigmented collarette, and eye color (mainly green). Additionally, higher freckle and nevus counts were observed in participants with more facial freckles and cutaneous nevi and were positively associated with each other. After adjustment, a positive significant association was identified between the presence of iris freckles and genetic variants in the *IRF4*, *HERC2*, and *OCA2* genes, as well as *SLC45A2*, although only in females. The prevalence of iris nevi was significantly lower compared to freckles. The presence of iris nevi also showed positive associations with genetic variants in *IRF4* and *HERC2*, plus *TYR* in brown-eyed individuals only. No association was identified between *MC1R*, the major cutaneous freckle gene, and the presence of iris freckles or nevi.

**Conclusions:**

The genetic basis of iris freckles and nevi reveals associations with well-known pigmentation genes (particularly *IRF4*), as well as eye color, sex, and age. These findings contribute to our understanding of iris pigmented benign lesions and their potential implications in conditions such as uveal melanoma, age-related macular degeneration, or solar damage.

The human iris is comprised of several distinct anatomical structures, each contributing to its unique pattern. It is composed of five different layers. The innermost layer, known as the iris pigmented epithelium, consists of densely packed cuboidal melanocytes that contain abundant melanin.^1^ This layer is present in all healthy humans and does not contribute to iris color or structure variations. Situated above the iris pigmented epithelium are two muscular layers—the sphincter and dilator muscle layers—which also do not play a significant role in iris anatomical diversity.^1^ Moving outward from the innermost part of the iris, the next layer is the stromal layer, followed by the anterior border layer. These two layers play the most significant role in determining individual differences in iris characteristics.^2^ The stromal layer is a complex intertwined network of melanocytes, immunological cells, and mostly fibroblasts and collagen fibers, whereas the anterior border layer, the most external, regularly contains fibroblasts and, especially in dark-eyed individuals, melanocytes.^3^ Also contributing substantially to the differences in iris color and structure is the presence of a pigmented collarette or peripupillary ring.^4^ The iris collarette is the middle portion ring of the iris, found between the pupil and the ciliary zone; it is slightly thicker than the rest of the iris and often is more darkly pigmented.^1^^,^^5^

Common pigmented iris lesions or spots, areas of darker pigmentation in the anterior border layer, may present as superficial freckles (also known as ephelides) or as deeper nevi.^6^ Pigmented spots generally smaller than 2 mm, flat, and not distorting the underlying stromal layer are classified as freckles, which occur in 40% to 70% of healthy adults, depending on the population. Iris nevi are generally larger and deeper benign lesions that grow downward and deform the iris stroma; they are found in 4% to 6% of adults and are typically solitary and better defined.^2^ Both freckles and nevi are more common in individuals of European ancestry and appear predominantly in the lower half of the iris.^7^^,^^8^

It has been suggested that these pigmented iris spots may influence the perception of overall eye color.^9^^,^^10^ This is especially important in the field of forensic science, where, through the use of a DNA sample, accurate eye color predictions are frequently carried out.^11^ Thus, a better understanding of the genetic basis of iris surface pigmented spots could lead to more precise eye color prediction algorithms.

Additionally, some of these iris pigmentary features have been described as risk factors of different specific disorders and clinical conditions. For example, pigmented lesions on the iris have been directly correlated with cutaneous cancers,^12^ including melanoma^13^; age-related macular degeneration^14^; and chronic skin sun damage.^15^ Moreover, although a recent study found no correlation between iris pigmented lesions and a higher risk of uveal melanoma,^6^ earlier findings, though much older, have suggested a direct association.^16^

Because these types of benign pigmented lesions of the iris may hold relevance for multiple biomedical scientific disciplines such as forensics, ophthalmology, or dermatology and possess increasing clinical significance, it has become essential to enhance our understanding of the global prevalence and genetic foundation of these traits.^7^^,^^13^ Although there are already some works in the scientific literature that have tried to study the genetic basis of these common pigmented lesions,^7^^,^^13^^,^^17^ none so far has focused on the genetic differences between iris freckles and iris nevi. In our study, we made use of a slit lamp to distinguish between these two different ocular pigmented entities and to describe the genetic basis of both types of iris pigmented spots for the first time, to our knowledge.

Finally, most genetic studies on iris pigmented lesions up to now have focused on populations of northern European origin, and these same studies have revealed that there are significant differences in the distribution of most iris traits among distinct populations.^18^ As far as we are aware, this is the first work on the genetics of iris pigmented spots performed on a Mediterranean population.

In short, in this study we aimed to (1) characterize and describe differences in iris pigmented lesions across the Spanish population; (2) look at correlations among the different pigmentary traits, including iris freckles, iris nevi, iris color and collarette, as well as sex and age; and (3) examine the genetic markers associated with these iris pigmented features in our Spanish population of Mediterranean origin.

## Materials and Methods

This study received ethical approval from three different Ethics Committees: the Clinical Research Ethics Committee of the Castellón General University Hospital, the Clinical Research Ethics Committee of the Castellón Province Hospital, and the Ethics Committee for Research Integrity of the Jaume I University of Castellón. It was conducted in full compliance with the tenets of the Declaration of Helsinki. All participating individuals provided written informed consent.

### Study Population

A total of 1014 individuals volunteered between April 2022 and August 2023 for ophthalmological examination, iris photographs, and genetic analysis (referred to as the genetic cohort). These participants were recruited from the Ophthalmology Units of the Castellón General University Hospital and the Castellón Province Hospital, Castellón, Spain. Strict exclusion criteria were applied to ensure the validity and reliability of the collected data. Participants over 90 years were excluded, as very elderly individuals may present with age-related changes in the iris, introducing potential biases in the data. Furthermore, volunteers who were related to one another were excluded to prevent potential influences from familial genetic factors. In addition, none of the participants had a serious ocular pathology related to pigmentation of the retina or iris or to the anatomy of the iris. Participants who had undergone glaucoma treatment were also excluded. Also, participants under 18 years of age were omitted from the study for ethical reasons.

### Separate Demographic Survey of the General Spanish Population

The demographic and phenotypic characteristics of the 1014 participants of the genetic study are not representative of the general Spanish population, as a preselection was made for patients with green and blue eyes. The rationale behind this was to acquire a collection of participants where blue, green, and brown eye color had approximately similar frequencies, so the study could have stronger statistical power, as blue and green eye color frequencies in Spain are relatively low.

To address this limitation, a separate demographic survey was conducted involving all consecutive patients (completely different individuals to the ones mentioned above) who visited the hospital consultation of the same ophthalmologist for 24 months (from September 2022 to August 2024), although the exclusion criteria were the same as above. This allowed for an estimation of the frequencies of the eye characteristics under study—iris freckles and nevi, eye color and pigmented collarette—on the broader general Spanish population. For each participant, detailed observations were recorded, including eye color, presence/absence of a pigmented collarette, and the presence and number of iris freckles and nevi. No iris photographs or buccal samples for genetic analyses were taken in this cohort. This separate demographic survey (referred to as the demographic cohort) included a total of 1303 individuals and truly represents the average iris pigmentary features of the general Spanish population (Table 1).

**Table 1.** Distribution of Iris Pigmentary Characteristics in the Demographic and Genetic Cohorts

Trait	Phenotype	Demographic Cohort (*n* = 1303), *n* (%)	Genetic Cohort (*n* = 1014), *n* (%)
Eye color	Brown	918 (70.5)	339 (33.4)
	Green	236 (18.1)	339 (33.4)
	Blue	149 (11.4)	336 (33.2)
Iris freckles	Present	605 (46.4)	666 (65.7)
	One to three	301 (23.1)	290 (28.6)
	Four to 10	177 (13.6)	222 (21.9)
	More than 10	127 (9.7)	154 (15.2)
	Absent	698 (53.6)	348 (34.3)
Iris nevi	Present	63 (4.8)	139 (13.7)
	One	51 (3.9)	118 (11.6)
	More than one	12 (0.9)	21 (2.1)
	Absent	1240 (95.2)	875 (86.3)
Pigmented	Present	407 (31.2)	651 (64.2)
collarette	Absent	896 (68.8)	363 (35.8)

### Data Collection

All participants completed a comprehensive questionnaire capturing information on age, sex, medical history (including systemic and ocular conditions), and current treatments, both systemic and ophthalmologic. The questionnaire also collected detailed information regarding visible dermatological features, including the presence of freckles on the face and nevi in other body areas, both phenotypic characteristics that may be linked to genetic differences in the pigmentation of the iris.

### Iris Imaging

**Figure 1.**
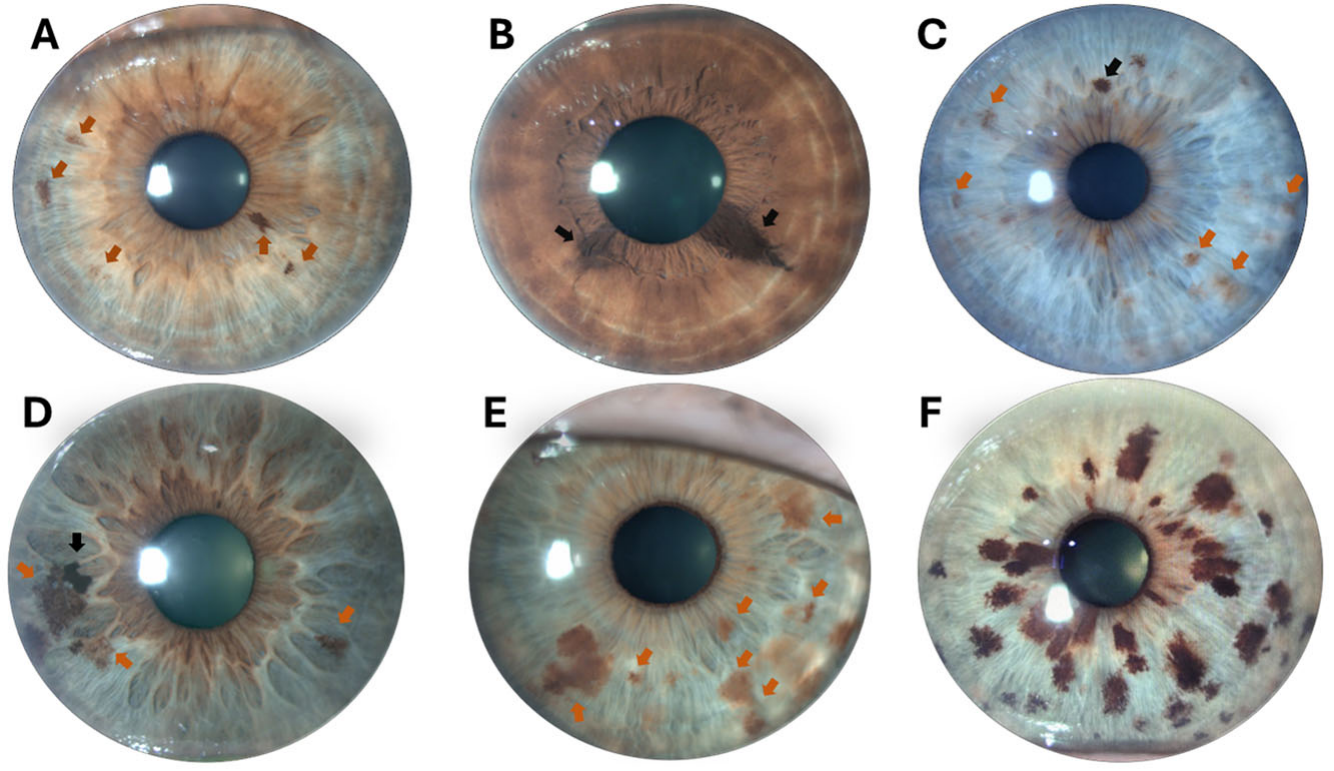
Digital images with examples of the different pigmentary elements found in the iris from six participants. (**A**) Freckles on a green eye with pigmented collarette. (**B**) Nevi on a brown eye. (**C**) Freckles (*orange arrows*) and nevus (*black arrow*) on a blue eye. (**D**) Freckles (*orange arrows*) and nevus (*black arrow*) on a green eye. (**E**) Multitude of iris freckles on a green eye. (**F**) An extremely rare case of an iris with a very large amount of nevi.

Detailed high-resolution photographs of the iris were obtained from all participants using a slit-lamp biomicroscope (Topcon Corporation, Tokyo, Japan) and the associated software Topcon IMAGEnet. This allowed for real-time, simultaneous observation of both irises. During this assessment, photographs were taken of both eyes to document and categorize the phenotypic features of each iris, including iris freckle and nevus count, eye color (blue, green/intermediate, brown), and presence/absence of pigmented collarette (Fig. 1). Images from the eye exhibiting the greater number of phenotypic features of interest (e.g., freckles or nevi) were selected for further evaluation. In instances where both irises showed comparable features, the left iris was used as the standard reference for analysis.

To ensure test–retest reliability, the same ophthalmology specialist reassessed a representative sample of 500 iris images in a blinded manner 6 months after the initial evaluation, without reference to the initial assessment. Thus, two assessment approaches, performed by the same ophthalmologist, were employed: (1) direct observation of the iris under the slit lamp at the time of the initial examination, and (2) subsequent analysis of the images captured during the first evaluation. There was complete concordance between the assessment of iris color conducted during each participant's visit and the subsequent interpretation of digital images 6 months later. In addition, there were very high correlation coefficients of reliability for pigmented collarette (Cronbach alpha of 0.95), iris freckles (Cronbach alpha of 0.88), and iris nevi (Cronbach alpha of 0.92), guaranteeing good data reliability. The use of slit-lamp examination by the expert ophthalmologist enabled precise differentiation between iris nevi and iris freckles, a distinction that has often been overlooked in prior studies.

In addition, an analysis was also carried out on the topography of the iris freckles and iris nevi to evaluate whether there were differences in terms of pigmented spot location within the iris. To do this, 456 iris photographs from different participants were evaluated for the number of freckles or nevi and the four quadrants of the iris in which they were located. In order to ensure the homogeneity of the data, all iris photographs, iris pigmentation features assessments, and questionnaire collections were performed and supervised by the same ophthalmology specialist.

### Biospecimen Collection

Genomic DNA was extracted from buccal samples collected from each participant using sterile swabs, a non-invasive method, to facilitate genomic analysis. DNA extraction was performed using the QIAamp DNA Mini Kit (QIAGEN, Hilden, Germany), following the manufacturer's recommended protocol. The isolated genomic DNA was subsequently processed for analysis.

### SNP Selection and Genotyping

A total of 37 single nucleotide polymorphism (SNP) markers were selected for genotyping based on their previously established or suggested associations with iris pigmented spots and/or eye color,^11^^,^^13^^,^^17^^,^^19^^,^^20^ as well as cutaneous freckling.^21^^–^^25^ These markers included the following: (1) SNPs mainly involved in eye color and/or presence of pigmented collarette in the *HERC2* gene (rs12913832,^11^ rs1129038,^19^ rs7183877,^20^ rs1667394,^20^ and rs916977^20^), *OCA2* (rs1800407,^11^ rs4778138,^20^ rs7495174,^20^ rs4778241,^20^ rs8024968,^20^ and rs1375164^20^), *SLC24A4* (rs12896399^11^ and rs4900109^17^), *SLC45A2* (rs16891982^11^), *IRF4* (rs12203592^11^), *TYR* (rs1393350,^11^ rs1042602,^22^ and rs1126809^19^), *TYRP1* (rs1408799,^19^ rs62538956,^19^ and rs13297008^19^), *HERC1* (rs11630290^17^), and *ARFIP2* (rs2253104^20^); (2) SNPs mainly involved in ocular and/or cutaneous freckling in *IRF4* (rs12203592^14^^,^^21^), *TYR* (rs1393350^22^ and rs1042602^22^), *DEF8* (rs8049897^24^), *ASIP* (rs4911442^25^), *BNC2* (rs2153271^23^), and *MC1R* (rs1805005,^21^ rs1805006,^21^ rs2228479,^21^ rs11547464,^21^ rs1805007,^21^ rs1110400,^21^ rs1805008,^21^ rs885479,^21^ and rs1805009^21^); and, finally, (3) SNPs involved in the presence of iris nevi, including *HERC1* (rs11630290^17^) and *XKR6* (rs7822804^17^) and the chromosome 12q24.21 region (rs11067162^17^). Notice that some SNPs in the *IRF4* and *TYR* genes are listed in both the eye color and the freckling categories, and the *HERC1* SNP is listed in the eye color and the nevi categories. This SNP selection aimed to investigate the genetic underpinnings of iris pigmentary characteristics, utilizing genetic markers with a history of association with iris and/or cutaneous pigmentation traits in populations of European ancestry.

SNP genotyping was conducted by the Spanish National Genotyping Center (CeGen-PRB2, Santiago de Compostela, Spain) as a contract service. Briefly, SNP genotyping was achieved by using the MassARRAY iPLEX Gold technology, according to manufacturer's protocol (Sequenom, San Diego, CA, USA). Assays were implemented in 384-well plates, together with a negative control and three Coriell samples for quality control. Genotyping accuracy was also assessed by adding three DNA duplicates per plate, producing 100% consistent replication outcomes.

### Characterization of Iris Pigmentary Features

Iris freckles were quantified using a four-grade system, allowing for precise measurement of their presence and distribution within the iris surface. The grading system was as follows: Grade 0, absence of freckles; Grade 1, one to three freckles; Grade 2, four to 10 freckles; and Grade 3, more than 10 freckles. Likewise, iris nevi were also classified into categories, in this case a three-grade scale was used: Grade 0, absence of nevi; Grade 1, one nevus; and Grade 2, more than one nevus. These two variables, iris freckles and iris nevi, were considered as ordinal variables in all statistical analyses. The ring was analyzed based on the amount of melanin surrounding the pupil—that is, the pigmented collarette. Two main categories were established: absence and presence of a pigmented collarette (Fig. 1). Finally, eye color was classified into the three classical groups discernible to the naked eye: blue (including gray), green/intermediate (including dark green and amber), and brown (Fig. 1).

### Statistical Analysis

All statistical analyses were performed using R 4.2.2 (R Foundation for Statistical Computing, Vienna, Austria) and SPSS Statistics 29.0.0.0 (IBM, Chicago, IL, USA). Multivariate logistic regression was utilized to investigate potential associations between various iris characteristics and the genetic variants analyzed in the study, with the genotype of each participant included as a covariate. Additionally, associations between the main variable and the predictor were evaluated by adjusting for different covariates, with reference to the different subgroups analyzed (in this case, only males or only females, only blue eyes, only green eyes, or only brown eyes). Independently of the main logistic regression model comprised of iris freckles or nevi as outcome and all the other variables as covariates (the SNPs, eye color, sex, age, and collarette), five other models were generated in which only females, only males, only blue eyes, only green eyes, and only brown eyes were taken into account. For each analysis, the risk associated with being homozygous for the ancestral allele, homozygous for the derived allele, or heterozygous for each SNP was estimated. In the case of the *MC1R* gene, due to the relatively low frequency of all nine SNPs genotyped, the number of mutations for all nine most common *MC1R* SNPs was accumulated in a score from 0 to 5, where 0 denoted an individual without *MC1R* mutations; 1, a participant exhibiting only one *MC1R* mutation; and so on. Five was the highest number of *MC1R* mutations present in any individual (there were no participants with six or more *MC1R* mutations).

The association between genotype and iris pigmentation was expressed as odds ratios (ORs) with corresponding 95% confidence intervals (CIs). For the logistic regression model and OR calculations, both the freckle and nevi categories were clustered into two groups: absence or presence; that is, the categories mentioned above of one to three freckles, four to 10 freckles, and more than 10 freckles were collapsed into a “freckle presence” category, and the same for iris nevi. The statistical significance of the associations was determined by the *P* value for each test, with Bonferroni correction applied to account for multiple comparisons. A threshold of *P* < 0.05 was used to identify statistically significant correlations between iris features and between these features and the participants’ genotypes.

Differences in the frequency of iris features across the participants were assessed using the χ^2^ test and by analyzing the expected frequencies versus the observed frequencies in each case. The association of iris freckles and nevi with age, the only continuous variable of the study, was performed using ANOVA.

## Results

### Demographic Survey: Phenotypic Characteristics of the General Spanish Population

This demographic study included 1303 volunteers, with an equal number of males and females (51% and 49%, respectively), and with an average age of 66 years. In terms of eye color, 71% had brown eyes, 18% had green/intermediate eyes, and 11% of the general Spanish population had blue eyes (Table 1), in line with previous studies.^26^^–^^28^ Regarding iris freckles and nevi, 47% of the general Spanish population had at least one iris freckle, whereas 53% had none. About half of the iris freckled population had one to three iris freckles (23%), up to 14% had four to 10 freckles, and 10% had more than 10. Iris nevi were considerably more infrequent, with only 4.8% of the general Spanish population presenting one iris nevus or more (3.9% with only one iris nevus and 0.9% with more than one). Regarding the pigmented collarette, 32% of the participants exhibited a pigmented collarette. The remaining 68% of the general Spanish population did not show a pigmented collarette (Table 1).

### Genetic Study: Data and Association Analyses From the Genetic Cohort

The genetic study included 1014 volunteers. The average age of these participants was 58 years, with a slightly higher proportion of females (59%) than males (41%). In the genetic study cohort, 339 individuals had brown eyes (33.4%), another 339 individuals had green/intermediate eyes (33.4%), and 336 individuals had blue eyes (33.2%) (Table 1). Additionally, 63% of the volunteers in the genetic cohort exhibited a pigmented collarette, and 37% did not. In terms of iris freckles, 34% did not display iris freckles, 29% had between one and three freckles, 22% had between four and 10, and 15% had more than 10. Regarding iris nevi, 86% showed no nevi, 12% had one nevus, and 2% had more than one (Table 1).

**Figure 2.**
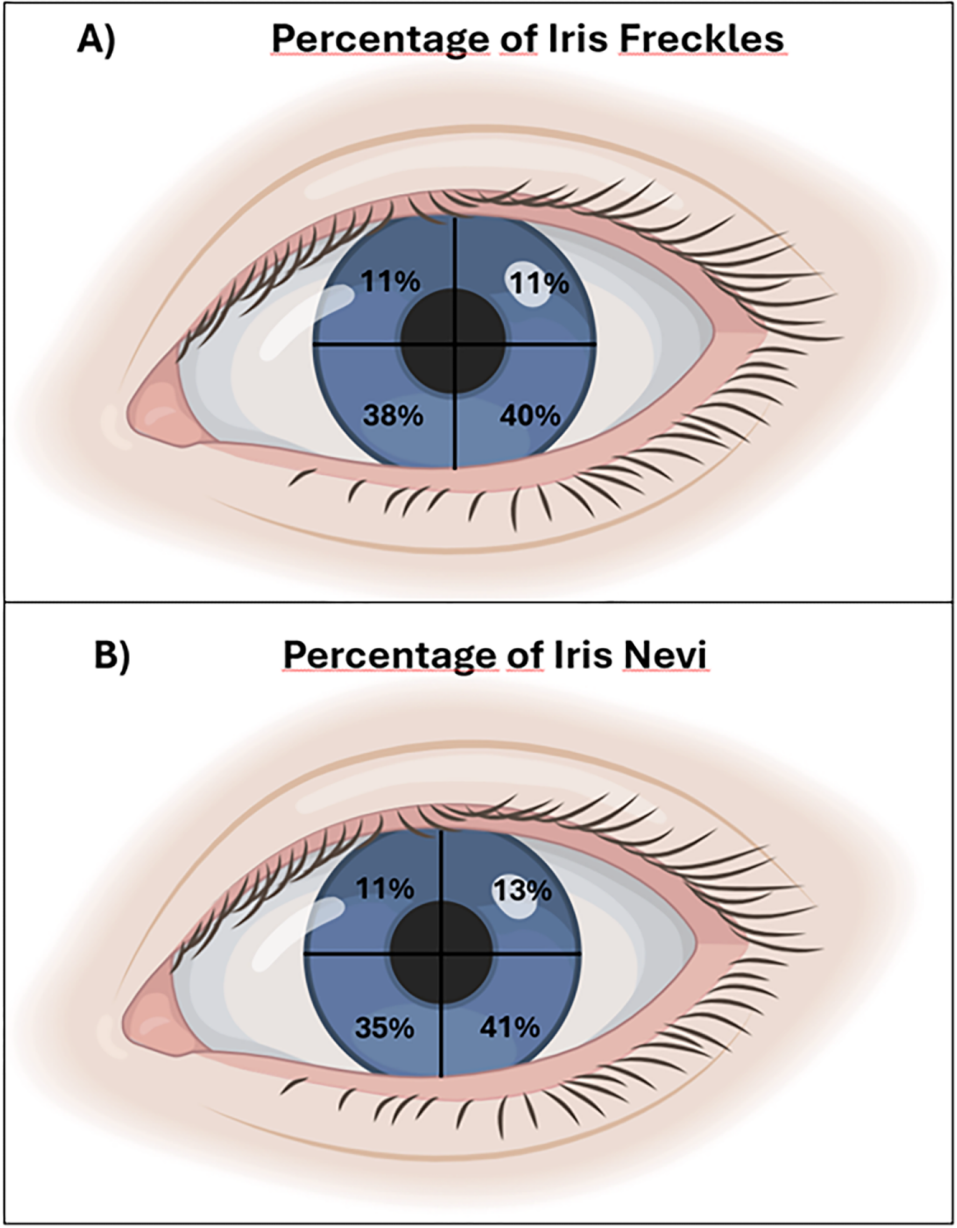
Graphical representation of a left eye showing the topographic distribution of the percentages of the different pigmentary spots in the iris by quadrants. (**A**) Iris freckles. (**B**) Iris nevi. The lacrimal point corresponds to the nasal meridian, and the opposite corresponds to the temporal meridian.

As previously observed,^7^^,^^8^ the majority of freckles and nevi were located in the lower half of the iris (Fig. 2). The percentages of freckles in each quadrant were 40% in the lower temporal quadrant, 38% in the lower nasal quadrant, 11% in the upper temporal quadrant, and 11% in the upper nasal quadrant. Regarding nevi, the percentages in each quadrant were 41% in the lower temporal quadrant, 35% in the lower nasal quadrant, 13% in the upper temporal quadrant, and 11% in the upper nasal quadrant (Fig. 2). Thus, significant differences were observed when comparing the upper half with the lower half of the iris (*P* = 1.08 × 10^−70^ for iris freckles and *P* = 2.09 × 10^−6^ for nevi). All results and associations presented in the Results section from here onward refer to this genetic cohort.

### Association of Iris Freckles and Nevi With Other Pigmentation Features

As reported previously,^2^ the number of both types of pigmented lesions in the iris was observed to increase with age in our study (*P* = 5.88 × 10^−8^ for freckles and *P* = 5.72 × 10^−5^ for iris nevi) (Table 2). Moreover, higher counts of especially iris freckles (*P* = 4.73 × 10^−4^) as well as iris nevi (*P* = 0.024) were found in females compared to males (Table 2). Additionally, individuals with a greater number of cutaneous nevi exhibited a higher count of iris freckles (*P* = 1.35 × 10^−4^) and iris nevi (*P* = 0.032). Similarly, a notable positive association was also demonstrated between facial freckles and iris freckles (*P* = 1.44 × 10^−3^) and iris nevi (*P* = 7.11 × 10^−3^), in contrast to previous studies.^13^

**Table 2.** Association of Iris Freckles and Iris Nevi to Other Phenotypic Characteristics

	Iris Freckles, *n* (%)					Iris Nevi, *n* (%)			
Variable/Trait	Absence	1–3	4–10	>10	*P* ^*^	Absence	1 Nevus	>1 Nevus	*P* ^*^
Average age (y)	54.2	56.2	61.0	62.4	**5.88 × 10^−^^8^**	57.0	63.1	69.2	**5.72 × 10^−^^5^**
Sex									
Female	154 (25.8)	175 (29.3)	136 (22.7)	133 (22.2)	**4.73 × 10^−^^4^**	503 (84.1)	78 (13.0)	17 (2.9)	**0.024**
Male	142 (34.1)	117 (28.1)	103 (24.8)	54 (13.0)		372 (89.4)	40 (9.6)	4 (1.0)	
Eye color									
Blue	107 (31.8)	82 (24.4)	95 (28.3)	52 (15.5)	**1.36 × 10^−^^24^**	288 (85.7)	42 (12.5)	6 (1.8)	**0.020**
Green	66 (19.5)	99 (29.2)	90 (26.5)	84 (24.8)		280 (82.6)	47 (13.9)	12 (3.5)	
Brown	175 (51.6)	109 (32.2)	37 (10.9)	18 (5.3)		307 (90.6)	29 (8.5)	3 (0.9)	
Pigmented collarette	167 (25.7)	191 (29.3)	158 (24.3)	135 (20.7)	**9.6 × 10^−^^3^**	550 (84.5)	88 (13.5)	13 (2.0)	**0.027**

Bold indicates significant results.

* *P* values are for the χ^2^ analysis.

Both iris freckles and nevi turned out to be significantly associated with iris color (*P* = 1.36 × 10^−24^ for iris freckles and *P* = 0.020 for nevi). High values of iris freckles and nevi were more common in individuals with green eyes (Table 2), whereas lower values were typically observed in those with brown eyes for both iris freckles and iris nevi (Table 2). Regarding blue eyes, they seem to have behaved in a relatively neutral way with respect to iris freckles and nevi, with results halfway between green and brown eyes. At the same time, we found that both iris freckles (*P* = 9.6 × 10^−3^) and iris nevi (*P* = 0.027) were positively correlated with the presence of a pigmented collarette (Table 2). Finally, the relationship between iris nevi and iris freckles exhibited a strong positive correlation. Statistical analyses indicated that high counts of iris freckles were associated with a greater number of iris nevi (*P* = 5.07 × 10^−16^).

### Association of Iris Freckles and Nevi With Genetic Markers

Concerning the correlation between iris freckles or nevi with the genetic markers genotyped, an association was identified involving five key genes related to skin and iris pigmentation: *IRF4* (rs12203592), *HERC2* (rs12913832), *OCA2* (rs8024968), *SLC45A2* (rs16891982), and *TYR* (rs1393350), after adjustment for all confounding variables.

Regarding the number of iris freckles, several genes were positively associated (Table 3). *IRF4* (the derived T allele of rs12203592) was by far the most significantly associated gene (*P* = 3.04 × 10^−15^), and *HERC2* (the G allele of rs12913832; *P* = 7.18 × 10^−5^), the main eye color gene, came second in importance (after controlling for all confounding variables, including eye color). The associations of SNPs rs8024968 (the derived T allele) in *OCA2* (*P* = 4.96 × 10^−4^) and the C allele of rs16891982 in the *SLC45A2* gene (*P* = 7.33 × 10^−3^), although weaker, were also significant, but in the case of *SLC45A2* the association was significant only for females. Importantly, no relationship was found between variants of the *MC1R* gene, the main skin freckle gene and a notable skin color gene, and the presence of iris freckles.

**Table 3.** Multivariate Logistic Regression Testing Association of the Number of Iris Freckles and Nevi With Genetic Variants (Adjusted for Age, Sex, Eye Color, and Pigmented Collarette)

		Iris Freckles		Iris Nevi	
Gene	SNP	OR (95% CI)	*P* ^*^	OR (95% CI)	*P* ^*^
*IRF4*	rs12203592	23.6 (17.1–32.5)	**3.04 × 10^−^^15^**	2.85 (2.47–3.29)	**1.68 × 10^−^^5^**
*HERC2*	rs12913832	3.27 (2.82–3.81)	**7.18 × 10^−^^5^**	3.28 (2.82–3.81)	**3.56 × 10^−^^4^**
*OCA2*	rs8024968	3.03 (2.56–3.57)	**4.96 × 10^−^^4^**	0.98 (0.64–1.51)	0.62

Only significant SNPs for the main logistic regression model are shown.

Bold indicates significant results at the Bonferroni threshold.

* *P* values are for the multivariate logistic regression association analysis.

In the case of the number of iris nevi, only positive associations with *IRF4* (T allele of rs12203592; *P* = 1.68 × 10^−5^) and *HERC2* (G allele of rs12913832; *P* = 3.56 × 10^−4^) were identified after controlling for all confounding variables (Table 3). For iris nevi, an association with brown eyes only was also found for the *TYR* gene (A allele of rs1393350; *P* = 2.43 × 10^−5^). Note that *MC1R* also lacked association with iris nevi. A previously described strong association of iris nevi with *HERC1*^17^ was absent in our study. Variants for the three major SNPs found associated with iris freckles and nevi (in the *IRF4*, *HERC2*, and *OCA2* genes), their risk alleles, and the minor allele frequencies (MAFs) for the different freckle and nevus subgroups (absence and presence) are given in Table 4.

**Table 4.** MAFs of the Main SNPs Associated With Iris Freckles and Nevi

				Iris Freckles		Iris Nevi	
Gene	SNP	Risk Allele	MAF, Genetic Cohort^*^	Absence (*n* = 348)	Presence (*n* = 666)	Absence (*n* = 875)	Presence (*n* = 139)
*IRF4*	rs12203592	T	0.17	0.11	0.20	0.16	0.27
*HERC2*	rs12913832	G	0.49	0.43	0.54	0.47	0.51
*OCA2*	rs8024968	T	0.14	0.18	0.12	0.14	0.15

Only significant SNPs are shown.

* Note that the MAF of the genetic cohort is not representative of the general Spanish population.

### Association of Facial Freckles and Cutaneous Nevi With Other Variables and Genes

Concerning facial freckles, an inversely proportional relationship with age was verified (*P* = 0.028), with younger people showing facial freckles more often. This contrasts with iris freckles, which are significantly more common in elderly people. Also, a correlation with sex was demonstrated, showing that females are more likely to develop facial freckles (*P* = 0.001). In the case of skin nevi, elderly individuals tend to have higher counts of nevi, with the number increasing with age (*P* = 5.7 × 10^−6^). Additionally, there is also a sex bias, with females also having a higher number of cutaneous nevi compared to males (*P* = 8.9 × 10^−6^).

In terms of genetic correlations, facial freckles turned out to be significantly associated with the *IRF4* gene (rs12203592; *P* = 1.68 × 10^−28^), *BNC2* (rs2153271; *P* = 8.21 × 10^−4^), *DEF8* (rs8049897; *P* = 8.84 × 10^−4^), and especially *MC1R* variants (*P* = 2.92 × 10^−34^), as previously described in genetic studies of cutaneous freckling.^21^^,^^29^^,^^30^ Regarding cutaneous nevi, significant associations were identified between the *IRF4* gene (*P* = 4.19 × 10^−14^) and the *MC1R* gene (*P* = 8.64 × 10^−4^), also as previously published.^31^^,^^32^

## Discussion

This work highlighted the relationship between iris pigmented benign lesions and both age and sex. Both types of iris pigmented spots (freckles and nevi) can enlarge over time, and, although they can be found across all age groups, older individuals are more predisposed to both types.^2^^,^^15^^,^^17^ However, a recent study found no correlation between pigmented spots and age or sex,^7^ which greatly contrasts with our findings. In our study, there was a clear predominance of freckles associated with increasing age, along with a significant increase in the presence of iris freckles in females. In fact, this is the first study that describes a clear positive association between iris freckles or nevi and female sex (Table 2). Numerous previous studies have already described positive female sex biases in human pigmentation, including skin pigmentation, cutaneous freckling, and eye color.^33^^–^^36^ Also, in this study we have described an association between a well-known pigmentation gene, *SLC45A2*, and iris freckling only in females, suggesting that there may also be sex-specific genetic effects operating in the pigmentation of the iris.^33^^,^^34^

In addition, this work shows that the supraorbital arch and the upper lid appear to protect the iris against freckle or nevus formation, as a strong statistical association between iris freckles or nevi and the lower area of the iris was found. In contrast, our study did not find significant evidence to suggest that the nose has a similar effect, since we did not find significant differences between the temporal and the nasal halves (as opposed to some previous works).^7^

As expected, the *MC1R* gene, previously reported to lack association with iris color^37^ or uveal melanoma,^38^^,^^39^ has no bearing on iris pigmented lesion development. The role of *MC1R* in melanogenesis is probably not required in tissues that are not ultraviolet (UV) responsive, which is consistent with its lack of involvement in iris pigmentation.^37^ Iris melanocytes do not require *MC1R* regulation because their melanin production is independent of UV exposure.^37^ Thus, other regulatory molecular mechanisms may be in charge of controlling iris pigmentation. In any case, this study definitely demonstrated that *MC1R*, the major cutaneous freckle gene, does not mediate pigmented spot development in the iris.

Perhaps the most surprising finding of this study, compared to prior knowledge, is the strong positive association between green eyes and both iris freckles and nevi. In addition, for the first time, to our knowledge, we identified a relatively neutral relationship of blue eyes with both iris freckles and iris nevi. In contrast, brown eyes, as previously reported,^2^^,^^5^^,^^7^ are clearly negatively correlated with both iris freckles and nevi. Blue eyes exhibit low levels of melanin in the stroma, exposing the iris to more UV light.^1^^–^^3^ But, perhaps the genetic variants associated with blue eyes may also be reducing the overall activity of melanocytes, potentially lowering the chances of freckle and nevus formation despite high UV exposure. However, green eyes have an intermediate amount of melanin compared to blue and brown eyes. We hypothesize that this balance of melanin may provide some protection, but not as much as brown eyes, leaving green eyes both as susceptible to UV-induced melanocytic damage as blue eyes (and much more than brown eyes), and at the same time with increased melanocytic pigmentary activity compared to blue eyes—enough to be capable of generating pigmented lesions. But, this is just a suggestion; further work must be performed to corroborate these findings.

Likewise, the association detected between iris freckles or nevi and the presence of a pigmented collarette is likely due to the high prevalence of these pigmented rings in green eyes. In fact, Larsson et al.^17^ reported that a pigmented collarette was present in 91% of individuals having green or hazel eye color.

In terms of genetic association, the *IRF4* gene, an important human pigmentation gene^40^ and the second most important for the development of freckles in the skin,^21^^,^^29^ is by far the most significantly associated with both iris freckles and iris nevi in our study. The SNP in this gene, rs12203592, is located in an enhancer region that regulates *IRF4* function,^41^ and its derived T allele has already been associated with dark hair color, light eye color, cutaneous freckling, and decreased ability to tan.^31^^,^^42^ Here, we have described an association of the T allele of *IRF4* with iris freckles and with iris nevi. The four other genes identified in our analysis that also demonstrated significant association with iris freckles or nevi (*HERC2*, *OCA2*, *TYR*, and *SLC45A2*) have also been previously correlated to eye color,^11^ and all were significantly associated even after controlling for eye color, although *OCA2* and *SLC45A2* were associated with iris freckles only, and *TYR* only with iris nevi exclusively for brown eyes. It seems that the presence of the risk A allele of the *TYR* SNP rs1393350 appreciably contributes to iris nevi formation only in the presence of brown eyes, whereas its effect is perhaps overshadowed by *IRF4* and *HERC2* in the case of green/intermediate eyes. Interestingly, both rs12203592 in *IRF4* and rs12913832 in *HERC2*, the two most important genetic determinants of iris freckles and nevi found in this work, have also been recently identified as risk factors for developing uveal melanoma.^43^^,^^44^

This study also has several limitations. The candidate gene approach inherently focuses on preselected genes based on prior knowledge, potentially overlooking novel or unexpected genetic contributors. For iris nevi, in particular, a significant limitation is the probable omission of genes known to influence cutaneous nevi. Also, phenotypic classification challenges, such as accurately distinguishing between iris freckles and nevi or capturing subtle variations in iris pigmentation or color, may introduce bias.

The findings in this work highlight the potential of iris pigmented benign lesions as markers for identifying individuals at heightened risk for UV-related health conditions. Iris freckles and nevi are linked to an increased risk of uveal melanoma, skin cancers including cutaneous melanoma, and age-related macular degeneration, as well as chronic skin sun damage. Investigating their genetic bases is therefore becoming of biomedical and clinical value.

In short, this study sheds light on the complex relationships between iris pigmentation and its genetic underpinnings. It has established a clear positive association among age, green eye color, and the prevalence of both iris freckles and nevi. It also identified a novel positive association between iris pigmented spots and female sex, with a specific link between iris freckles in females and the pigmentation gene *SLC45A2* that suggests sex-specific genetic effects. From a genetic perspective, this study highlighted the *IRF4* gene as a key player associated with both iris freckles and nevi. This gene was identified as the major contributor to these traits, marking the first reported association between *IRF4* and iris nevi. Other eye pigmentation-related genes (*HERC2*, *OCA2*, *TYR*, and *SLC45A2*) are also implicated, even when controlling for eye color. Notably, *TYR* was exclusively linked to iris nevi in brown-eyed individuals. These findings not only deepen our understanding of the genetic basis of iris pigmented benign lesions but also point to potential new directions for studying eye pigmentation.
